# The Reliability and Validity of the Leg Lateral Reach Test in Adolescent Tennis Players

**DOI:** 10.5114/jhk/197429

**Published:** 2025-10-01

**Authors:** Elif Aygun-Polat, Sevim-Beyza Olmez, Zeynep Hazar

**Affiliations:** 1 Department of Physiotherapy and Rehabilitation, Faculty of Health Science, Ordu University, Ordu, Turkey.; 2 Department of Physiotherapy and Rehabilitation, Faculty of Health Science, Karamanoglu Mehmetbey University, Karaman, Turkey.; 3 Department of Physiotherapy and Rehabilitation, Faculty of Health Sciences, Gazi University, Ankara, Turkey.

**Keywords:** range of motion, athletic injuries, reproducibility of results

## Abstract

Regular assessment of clinical measurements of trunk rotation flexibility that are valid and reliable is necessary to minimize the risk of injury and optimize performance in tennis players who perform repetitive trunk rotation. The objectives of this study were to investigate: (1) the intra-rater, inter-rater, and test-retest reliability of the leg lateral reach test (LLRT) in tennis players; (2) the minimum detectable change in the leg lateral reach test in tennis players; and (3) the correlation between leg lateral reach and seated rotation tests in tennis players. Twenty-four adolescent tennis players were recruited for the study. To ascertain the reliability of the leg lateral reach test, two designated raters (rater 1 and rater 2) evaluated each participant with the test 5–7 days apart (session 1 and session 2). The leg lateral reach distances and the seated rotation angle from session 1 were correlated to evaluate the validity. The intra-rater and inter-rater analysis found almost perfect reliability (ICC ≥ 0.835, SEM ≤ 2.47 cm; ICC ≥ 0.881, SEM ≤ 2.02 cm, respectively). The correlation between the distance of the lateral leg reach for the dominant and non-dominant sides and the angle of seated rotation was fair (r 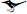 0.441 and 0.406, respectively). This study demonstrates the leg lateral reach test as a reliable and valid screening tool, accurately predicting the range of thoraco-lumbo-pelvic rotation in adolescent tennis players.

## Introduction

Tennis is defined by powerful, repetitive, and well-coordinated movements throughout the kinetic chain (Elliott, 2006; Kovacs and Ellenbecker, 2011). The entire body, particularly the trunk, is put under much strain by the physical demands of tennis (Rice et al., 2022). For a good shot in tennis, which is both an overhead and rotational sport, the high forces and energy produced are transported from the lower extremities to the upper extremities through the trunk (Campbell et al., 2014; Donatelli et al., 2012; Fleisig et al., 2003). Thus, the trunk's capacity to generate, absorb, and transfer force from the lower limbs to the upper body is essential for tennis players (Rice et al., 2022).

In tennis players, limited trunk rotation flexibility can lead to abnormal trunk motion patterns, which can cause upper extremity injuries as well as lower back pain and injuries (Aragon et al., 2012; Pontes-Silva et al., 2021). Previously, some authors have noted that the highest percentage of trunk injuries occurred among adolescent tennis athletes (13–17 years old), and injury data suggest that additional injury prevention programs in tennis are necessary (Hjelm et al., 2012; Jayanthi et al., 2015). Therefore, it is essential to evaluate and treat disorders in the fundamental building blocks of movement, such as trunk rotation flexibility, to minimize the risk of injury and optimize performance in tennis players (Rice et al., 2022).

The leg lateral reach test (LLRT) was recently developed by Kim et al. (2017) to assess the thoraco-lumbo-pelvic rotation range and was valid and reliable for physically active, healthy individuals. The test's low cost and simplicity are its advantages (Kim et al., 2017). Pontes-Silva et al. (2021) stated that the test is a reliable measure of thoraco-lumbo-pelvic rotation in individuals with nonspecific chronic low back pain and can be used as a prognostic tool to assess treatment effects on low back mobility in this population (Pontes-Silva et al., 2021). However, the measurement properties of the LLRT have yet to be systematically investigated for athletes who perform repetitive trunk rotation, such as tennis players. We hypothesized that the LLRT would be a reliable and valid measure in tennis players. The validity and reliability of the test for tennis players would help assess athletes' trunk rotation flexibility, compare different groups of athletes, and identify the exercise regimen required to enhance performance. Therefore, the objectives of this study were to investigate: (1) the intra-rater, inter-rater, and test-retest reliability of the LLRT in tennis players; (2) the minimum detectable change in the leg lateral reach test in tennis players; and (3) the correlation between leg lateral reach and seated rotation tests in tennis players.

## Methods

### Study Design

This cross-sectional study was conducted at the Ankara Tennis Club, Ankara, Turkey, between September 2023 and January 2024. The Gazi University Clinical Research Ethics Committee obtained ethical approval (protocol code: 2023-942; approval date: 27 March 2023). The Declaration of Helsinki's criteria were followed when conducting the study. All participants' parents signed written informed consent forms. The study was registered at the Clinical Trials Registry as NCT06025656.

### Procedures

The demographic data of participants were recorded. Two designated raters (rater 1 and rater 2) assessed each participant using the LLRT 5–7 days apart (session 1 and session 2) to determine the test's reliability. Each participant's leg reach distance was measured independently by both raters, skilled physiotherapists with more than five years of clinical experience, three times per session. The raters were blinded to each other's findings. By drawing lots, the examiners' order was decided upon at random. Before the second examiner tested the participants, they were given five minutes of comfortable relaxation. In session 1, seated rotation test assessments were conducted for each participant. A five-minute rest interval was provided between each test to reduce the potential effects of fatigue.

### Participants

Twenty-four licensed adolescent tennis players who had played tennis for at least two years were included. Volunteers were excluded if athletes (1) had a history of musculoskeletal injury or surgery in the last year that would prevent the tests, or (2) felt pain in the trunk and lower extremities during the tests. The participants' clinical and demographic characteristics are shown in Table 1.

**Table 1:** Characteristics of the participants.

Variables	Mean ± SD
Age, y	12.50 ± 1.76
Body height, cm	158.04 ± 12.67
Body mass, kg	46.22 ± 10.69
BMI, kg/m^2^	18.31 ± 2.28
Tennis experience, y	6.13 ± 1.89
Weekly training hours, h	14.58 ± 4.08
	n (%)
Sex Male Female	14 (58.3) 10 (41.7)
Dominant side Right Left	22 (91.7) 2 (8.3)

y, year; h, hour; SD, Standard Deviation

### Outcome Measures

#### Leg Lateral Reach Test

The leg lateral reach test was recently developed to assess the thoraco-lumbo-pelvic rotation range without technology. All participants completed practice trials to familiarize themselves with the experimental procedure before data collection. The LLRT started with the participants placing their arms at their sides. After that, they were instructed to rotate their trunks while raising the leg, which was being evaluated, and extending it across the other leg to make contact with a wooden bar. Participants were consistently encouraged to stretch their tested leg as far as possible along the length of the wooden bar and to ensure that both shoulders stayed in contact with the floor during each trial. A trial was deemed invalid and excluded from the analysis if the participant could not maintain contact between their foot and the wooden bar at the point of maximal reach for five seconds or if both shoulders were lifted off the ground. After completing three rounds of this test on both the right and left sides, each participant's average reach distance was determined and registered for further analysis (Kim et al., 2017).

#### Seated Rotation Test

The seated rotation test is a clinical test to assess trunk rotation flexibility. The participant was instructed to sit on a chair with his/her arms crossed over her chest, the feet together and touching the floor, the her body upright and erect. The participant was then asked to turn to the right as far as they could without causing any discomfort. The test was then repeated, with rotation to the left. The amount of rotation was measured with a goniometer. The average of three trials was used for subsequent analysis (Aragon et al., 2012).

### Statistical Analysis

#### Sample Size Calculation

The sample size was calculated using G*Power software, version 3.1.9.2. The intraclass correlation coefficient (ICC) amplitude of the confidence interval was 0.30, and a confidence coefficient of 0.95 was considered when calculating the sample size *a priori*. Following the Fleiss's study, the computation was conducted to identify moderate reliability (ICC = 0.75) (Fleiss, 2011). Consequently, an estimated minimum sample size of 24 individuals was determined. The Bonett's study was the basis for calculating the sample size (Bonett, 2002).

#### Data Analysis

Described statistics were computed to characterize the individuals' demographic features. The data's normality was assessed using the Shapiro-Wilk tests.

The intra-class correlation coefficient (ICC) test was used to calculate the intra-rater (ICC model 3,1), inter-rater (ICC model 3,2), and test-retest reliability (ICC model 2,3). Since the ICCs model 3 is considered suitable for raters or average ratings from the same set of raters, it was utilized to calculate the degree of intra-rater and inter-rater reliability (Portney and Watkins, 2009).

The test-retest reliability was calculated using the ICCs model 2, recommended for methodological research because the same raters recorded the test's mean scores during both test sessions. The test's mean scores were determined to improve the likelihood of reliability estimations and more accurate estimates of genuine scores (Domholdt, 2005; Portney and Watkins, 2009).

A two-way mixed model of the ICC (95% confidence interval [CI]) was used to examine intra-rater, inter-rater, and test-retest reliability (Weir, 2005). A reliability coefficient of > 0.80 was deemed "almost perfect", 0.60–0.79 was "substantial", 0.40–0.59 was "moderate", 0.20–0.39 was "fair", and 0.00–0.19 was "slight" (Landis and Koch, 1977).

The standard measurement error (SEM) was computed to estimate the systematic error in the initial measurement unit (cm) (Weir, 2005). The following formula was used to determine the minimal detectable change at the 95% confidence interval (MDC_95_) based on the test-retest reliability results: MDC_95_ = 1.96 × SEM × √2 (Portney and Watkins, 2009). The degree of correlation between the seated rotation angle and the leg reach distance was verified using the Spearman correlation coefficient. A correlation coefficient (r) greater than 0.75 denoted a "good to excellent" association, 0.50–0.75 a "moderate to good" association, 0.25–0.50 a "fair" association, and 0.00–0.25 a "little or no" association (Portney and Watkins, 2009). All statistical analysis was done using the SPSS v. 22.0 software (IBM Corp., 184 Armonk, NY, USA). All tests were conducted with a significance level of *p* < 0.05.

## Results

The LLRT values recorded by each examiner in the test and retest are shown in Table 2. The intra-rater analysis, as presented in Table 3, found reliability values to be almost perfect (ICC ≥ 0.835; SEM ≤ 2.47 cm). Similarly, Table 4 demonstrates that the inter-rater analysis also found nearly perfect reliability (ICC ≥ 0.881; SEM ≤ 2.02 cm).

**Table 2:** The results of the leg lateral reach test in participants according to the two examiners.

	Examiner 1		Examiner 2	
Tested Side	Test	Retest	Test	Retest
Dominant, cm Mean ± SD	83.10 ± 5.82	84.04 ± 6.38	83.12 ± 5.27	84.16 ± 6.47
Non-dominant, cm Mean ± SD	80.12 ± 5.85	81.06 ± 6.52	80.93 ± 5.98	81.7± 7.06

SD, Standard Deviation

**Table 3:** **The intra-rater reliability of the measurement of the leg lateral reach test in participants**.

Tested side	Rater	ICC_2,3_	95% CI	SEM (cm)	MDC_95_ (cm)
Dominant	1	0.835	0.624–0.928	2.47	6.84
	2	0.861	0.684–0.939	2.18	6.04
Non-dominant	1	0.898	0.768–0.956	1.97	5.46
	2	0.882	0.730–0.949	2.23	6.18

CI, Confidence Interval; ICC, Intraclass Correlation Coefficient; SEM, Standard Error of Measurement; MDC, Minimum Detectable Change

**Table 4:** The inter-rater reliability of the measurement of the leg lateral reach test in participants.

Variables	ICC_2,3_	95% CI	SEM (cm)	MDC_95_ (cm)
Test Dominant Non-dominant	0.881 0.883	0.724–0.949 0.733–0.949	1.91 2.02	5.29 5.59
Retest Dominant Non-dominant	0.929 0.945	0.836–0.969 0.875–0.976	1.71 1.59	4.73 4.40

CI, Confidence Interval; ICC, Intraclass Correlation Coefficient; SEM, Standard Error of Measurement; MDC, Minimum Detectable Change

The correlation between the distance of the lateral leg reach for the dominant and nondominant sides and the angle of seated rotation and was fair, as shown in Table 5 (r = 0.441 and 0.406, respectively).

**Table 5:** The correlation between the leg lateral reach and seated rotation tests.

Variables	Leg Lateral Reach Test	r	*p*
Seated Rotation Test	Dominant	0.441	0.031^*^
	Non-dominant	0.406	0.049^*^

r, Spearman correlation coefficient magnitude; ^*^ Significant correlation (p < 0.005)

## Discussion

Clinical measurements of trunk rotation flexibility that are valid and reliable can be used to identify tennis players who are more vulnerable to injury or to help clinicians assess the extent of functional recovery following an injury. This study indicates that the leg lateral reach test (LLRT) is a reliable and valid screening tool, accurately predicting the range of thoraco-lumbo-pelvic rotation in adolescent tennis players.

The findings of this study reveal almost perfect intra- and inter-rater reliability results, consistent with those reported in patients with chronic low back pain (Pontes-Silva et al., 2021) and healthy individuals (Kim et al., 2017). Those prior studies indicated intra-rater reliabilities with ICC values of ≥ 0.889 and 0.966 between sessions and inter-rater reliability with ICC values of ≥ 0.947 and 0.990 within the same session, respectively (Kim et al., 2017; Pontes-Silva et al., 2021). Similarly, intra- and inter-rater reliability in the present study ranged from 0.835 to 0.898 and 0.881 to 0.945, respectively. Despite almost perfect intra- and inter-rater reliability, these values are slightly lower than those found by Pontes-Silva et al. (2021) in a sample of patients with chronic low back pain, with an average age of 30.9 years, and by Kim et al. (2017) in a sample of healthy individuals in their early 20s. The present study’s sample was substantially younger, with an average age of 12.5 years. Thus, this difference may be attributed to population-specific performance differences in adolescents compared to adults due to their variations in the maturing thoracolumbar spine, such as musculoskeletal development and adipose tissue distribution (Malina et al., 2004). In addition, the inter-rater reliability within the session was slightly higher than the intra-rater reliability between sessions in this study. In line with this, the SEM result for inter-rater reliability was slightly lower than that for intra-rater reliability (2.02 cm and 2.47 cm, respectively), which may be explained by the variability in athletes’ performance across testing sessions. Apart from this difference, both reliability analyses showed very low SEM values, similarly to the study in which the test was developed (1.40–2.66 cm) (Kim et al., 2017). According to the results of this study, changes equal to or greater than 2–2.5 cm in measurements in adolescent tennis players should be interpreted as alterations exceeding those caused by statistical variation.

Establishing MDC values for thoracic rotation ROM can help discern meaningful changes beyond random variations (Haley and Fragala-Pinkham, 2006). A thoracolumbar angle change exceeding 6.84 cm measured over time in adolescent tennis players can be considered significant, offering clinicians and researchers a reliable benchmark. Furthermore, MDC of the LLRT has been determined among patients with chronic low back pain and healthy individuals (Kim et al., 2017; Pontes-Silva et al., 2021). While the MDC value in the healthy individuals (7.37 cm) aligned with this study (Kim et al., 2017), it exhibited a higher value in patients with chronic low back pain (16.04 cm), which may be attributed to the limitations imposed by the pain (Pontes-Silva et al., 2021). In conclusion, these MDC values suggest that the test is a valuable assessment tool for individuals with different activity levels.

The present study found a fair correlation (r = 0.44) between the leg lateral reach and seated rotation tests. Although the seated rotation test is a valid and reliable trunk rotation flexibility test with low SEM and MDC values, it focuses on segmental thoracic rotation ROM with the pelvis and lower limbs fixed (Johnson et al., 2012). As the LLRT assesses more global trunk rotation involving the lower extremity, it is anticipated that the measurements of trunk rotation derived from the seated rotation test will not significantly correlate with it. However, in contrast to the current study, the study in healthy individuals in their early 20s found a higher correlation value (r = 0.73) between leg lateral reach and thoraco-lumbo-pelvic rotation (Kim et al., 2017), which was assessed in the horizontal plane with 3D kinematic analysis. In this study, the seated rotation was assessed with a goniometer. Differences in measurement methods and the sample group may have led to lower correlations.

This study found that the LLRT is a simple and quick clinical assessment of thoraco-lumbo-pelvic rotation which can provide reliable and valid data on adolescent tennis players. Study limitations include the fact that participants were exclusively adolescents, potentially affecting the generalizability of these findings. Nevertheless, given that low back injuries are more prevalent in adolescent tennis players with a growing spine that has not yet reached its total neuromuscular capacity due to asymmetrical loads, frequent repetitions, and high training volume, we found it very beneficial to evaluate the LLRT in this population (Johansson et al., 2022; Rice et al., 2022). Another limitation is that the seated rotation test, employed in the validity assessment, does not mirror the rotation pattern of the LLRT, as it lacks pelvic and lower extremity rotation. However, the LLRT in adolescent tennis players exhibited fair validity, thus it can be accepted that this factor did not significantly affect the results.

Our study revealed that the LLRT is reliable and valid for assessing thoraco-lumbo-pelvic rotation in adolescent tennis players. As reduced trunk rotation ROM and asymmetry may put adolescent tennis players at risk for low back pain or injury, the LLRT can be a helpful tool for identifying athletes with limited trunk rotation and documenting changes related to treatment. This information is of significance for coaches, physical trainers, clinicians, and researchers. Further research is needed to enhance the leg lateral reach test's ability to detect the thoraco-lumbo-pelvic axial rotation range in athletes.

## Conclusions

This study demonstrates the LLRT as a reliable and valid screening tool, accurately predicting the range of thoraco-lumbo-pelvic rotation in adolescent tennis players. As reduced trunk rotation ROM and asymmetry may put adolescent tennis players at risk for low back pain or injury, the LLRT can be a helpful tool for identifying athletes with limited trunk rotation and documenting changes related to treatment.
